# INVESTIGATING THE DAILY RELATIONSHIP BETWEEN SUBJECTIVE AGE AND DAILY EVENTS

**DOI:** 10.1093/geroni/igae098.2106

**Published:** 2024-12-31

**Authors:** Patrick Klaiber, Theresa Pauly

**Affiliations:** Tilburg University, Tilburg, Noord-Brabant, Netherlands; Simon Fraser University, Vancouver, British Columbia, Canada

## Abstract

Subjective Age, also known as felt age, refers to individuals’ perception of their age compared to their chronological age. Daily fluctuations in subjective age are linked to daily affective experiences; feeling older than usual has been associated with experiencing more same-day stressors and higher negative affect. Feeling older than usual may indicate depleted psychosocial resources, increasing the likelihood of interpreting everyday situations as stressful and reacting more intensely to them. Conversely, feeling younger than usual may indicate greater psychosocial resources, leading to increased engagement in and responsiveness to positive events. Thus, this study investigated fluctuations in subjective age as a predictor of same-day event engagement (stressors and positive events) and affective responses to these events using 14-day diary data from a sample of 108 older Swiss adults (aged 65-92). On average, participants felt approximately 8 years younger than their chronological age, with significant day-to-day variability (ICC =.69). We found that on days when individuals felt older than usual, they reported more stressors and fewer positive events. Older subjective age was also associated with lower same-day positive affect, but also greater increases in positive affect when positive events occurred. Similarly, older subjective age was related to greater same-day negative affect, yet also to more pronounced decreases in negative affect when positive events occurred. However, stress reactivity, as indicated by stressor-related affect, was not associated with subjective age. These findings underscore the importance of subjective age as a dynamic psychological construct potentially shaping individuals’ daily affective experiences.

